# Interventions to address social connectedness and loneliness for older adults: a scoping review

**DOI:** 10.1186/s12877-018-0897-x

**Published:** 2018-09-15

**Authors:** Hannah M. O’Rourke, Laura Collins, Souraya Sidani

**Affiliations:** 1 Faculty of Nursing Level 3, Edmonton Clinic Health Academy, University of Alberta, 11405-87 Avenue, Edmonton, Alberta T6G 1C9 Canada; 2 Daphne Cockwell School of Nursing, Ryerson University, 350 Victoria Street, Toronto, ON M5B 2K3 Canada

**Keywords:** Loneliness, Social connectedness, Intervention, Scoping review, Older adult

## Abstract

**Background:**

Older adults are at risk for loneliness, and interventions to promote social connectedness are needed to directly address this problem. The nature of interventions aimed to affect the distinct, subjective concepts of loneliness/social connectedness has not been clearly described. The purpose of this review was to map the literature on interventions and strategies to affect loneliness/social connectedness for older adults.

**Methods:**

A comprehensive scoping review was conducted. Six electronic databases were searched from inception in July 2015, resulting in 5530 unique records. Standardized inclusion/exclusion criteria were applied, resulting in a set of 44 studies (reported in 54 articles) for further analysis. Data were extracted to describe the interventions and strategies, and the context of the included studies. Analytic techniques included calculating frequencies, manifest content analysis and meta-summary.

**Results:**

Interventions were described or evaluated in 39 studies, and five studies described strategies to affect loneliness/social connectedness of older adults or their caregivers in a qualitative descriptive study. The studies were often conducted in the United States (38.6%) among community dwelling (54.5%), cognitively intact (31.8%), and female-majority (86.4%) samples. Few focused on non-white participants (4.5%). Strategies described most often were engaging in purposeful activity and maintaining contact with one’s social network. Of nine intervention types identified, the most frequently described were One-to-One Personal Contact and Group Activity. Authors held divergent views of why the same type of intervention might impact social connectedness, but social contact was the most frequently conceptualized influencing factor targeted, both within and across intervention types.

**Conclusions:**

Research to test the divergent theories of why interventions work is needed to advance understanding of intervention mechanisms. Innovative conceptualizations of intervention targets are needed, such as purposeful activity, that move beyond the current focus on the objective social network as a way to promote social connectedness for older adults.

**Electronic supplementary material:**

The online version of this article (10.1186/s12877-018-0897-x) contains supplementary material, which is available to authorized users.

## Background

The interplay of health and contextual factors puts older adults at risk for loneliness [1], a negative feeling resulting from a perceived deficit in companionship, quantity or quality in one’s relationships with either an attachment figure or a community [2–4]. Health factors include chronic disease experiences that interfere with functioning, and cognitive decline resulting in communication impairments [5] or inability to remember significant others or the recent interactions with them [6]. Contextual factors relate to lacking a confidant [7], relocation to a care facility as well as loss of loved ones due to death [2, 7, 8], and spending too much time alone [7] or idle [8], which yields feelings of separation from others. Loneliness may also be influenced by the number and structure of one’s relationships (i.e. social network), frequency of interaction with others (i.e. social contact), or the types and amounts of assistance received from others (i.e. social support) [9, 10].

Lonely people are at risk for reduced health and well-being, including poor life satisfaction [11–15], depression [16, 17], low self-esteem [13], reduced hope [13], negative affect [18, 19], and impaired function in activities of daily living [9, 17, 18]. The opposite of loneliness, social connectedness, is a basic human need [20] that may influence health and wellbeing for older adults, more than social contact, network or support [9]. Social connectedness is a positive subjective evaluation of the extent to which one has meaningful, close, and constructive relationships with other individuals, groups, or society indicated by: (1) feelings of caring about others and feeling cared about by others, such as love, companionship or affection and (2) feeling of belonging to a group or community [1].

Interventions to promote social connectedness/reduce loneliness among older adults have been analyzed and described in previous literature reviews in concert with interventions aimed at the distinct, but related, concept of social contact/isolation [21–26]. Intervention types were described by Dickens et al. (2011) based on their component activities and included “activities (social or physical programmes), support (discussion, counseling, therapy or education), internet training, home visiting, and service provision” [21] (p. 5). Other reviews categorized interventions based on a combination of the activities offered and the mode of delivery (e.g. group or one-to-one) [22–25]. Cattan et al. (2005) [22] described group (including activities of educational input or social support), one-to-one (including activities of home visits or telephone contact to provide information, services, or support), service provision (e.g. transport, medical intervention), and community development (e.g. social activities) interventions. Hagan et al. (2014) described group (including activities such as cognitive enhancement workgroups, adult day centre attendance, and gender-based social groups) and one-to-one interventions (including activities like befriending and mentoring programs, animal-assisted therapy, use of the internet, and contact with family or friends using a variety of technological approaches) [24]. Cohen-Mansfield et al. (2015) described group (educational, shared activity, or specific therapy techniques) and one-to-one activities (educational, sensory technological aids, and specific therapy techniques) [25]. Windle et al. (2011) described one-to-one interventions (befriending, mentoring, and gatekeeping), group services (day-centre services and social group schemes), and wider community engagement (participation in existing activities, outreach programmes, or volunteer schemes) [26]. Similarly, Findlay et al. (2003) identified group (tele-conferencing or discussion/support groups) and one-to-one (telephone support and gatekeeper programmes that connect socially isolated older adults with support services) interventions, as well as service provision (community support services) and use of the Internet.

Because interventions aimed to affect loneliness/social connectedness and isolation/social contact among older adults were reviewed and analyzed together in these previous reviews [21–26], which interventions actually addressed loneliness/social connectedness for this population is unclear. Isolation/social contact and loneliness/social connectedness are different concepts, the former objective and the latter subjective: one may be alone (i.e. isolated) and still feel a sense of social connectedness, or be surrounded by people (i.e. have social contact) and feel lonely [4, 8, 27]. The components and activities of interventions designed to target loneliness/social connectedness may differ in important ways from those designed to reduce isolation/increase social contact [28]. Furthermore, how the interventions addressed either the indicators of loneliness/social connectedness or its possible influencing factors is unclear. Conceptualization of the mechanisms by which interventions address loneliness/social connectedness, as opposed to isolation/contact, is needed [27, 29], responding to calls for theory-based interventions that are problem-specific [21, 22]. The purpose of this scoping review was to identify and describe the number and features of interventions that were designed to address loneliness/social connectedness among older adults. The aims were to: (1) describe and clarify the interventions’ goals, components, activities, mode and dose of delivery and (2) shed light on the theoretical underpinnings of these interventions by assessing the extent to which their hypothesized mechanisms of effect mapped to factors that may influence social connectedness.

## Methods

To identify and describe the nature of strategies and interventions designed to affect loneliness/social connectedness for older adults, scoping review procedures were applied to systematically search, screen, extract and synthesize this broad body of literature [30, 31]. Typical of scoping review methodology, quality appraisal was not conducted since our aim was not to assess intervention efficacy [30, 31]. Scoping review results are particularly useful to inform the design of future systematic reviews, which would include a transparent and rigorous quality appraisal process [30, 31]. The results of the current scoping review will identify types of interventions that are sufficiently homogenous in their components, activities, and theoretical underpinnings and evaluated across multiple studies, warranting assessment of their efficacy in future systematic reviews. Articles included in this scoping review reported on the: (i) design of an intervention; (ii) protocol or results of a quantitative study to evaluate an intervention; (iii) or findings of a qualitative study that explored older adults’ or caregivers’ strategies to promote social connectedness.

### Search procedures

Six electronic databases—Psycinfo, Medline, Cumulative Index to Nursing and Allied Health Literature, Proquest Dissertations and Theses, Proquest Nursing, and Proquest Social Service Abstracts —were searched in July 2015 from inception. Search strategies, using keywords and database-specific subject headings, were designed to capture the concepts of ‘social connectedness’ (using terms identified in collaboration with a health sciences librarian like social connectedness, connectedness, lonely, or loneliness) and ‘intervention’ or ‘strategy’ (using terms identified from a previous systematic review [32] like therapy, treatment, strategy, or intervention studies). The results were exported to Refworks and duplicates removed prior to screening. See Additional file 1 for full details of database-specific search strategies.

### Inclusion/exclusion criteria

Broad selection criteria were used to be comprehensive. Articles were included if they: (i) described an intervention evaluated using any quantitative, qualitative or mixed methods design, or explored in a qualitative study the strategies used by older adults or caregivers to promote social connectedness; (2) identified loneliness/social connectedness or its indicators (i.e. feelings of caring for/about others or feelings of belonging) as a primary goal or outcome of the intervention or strategies; (3) targeted adults 55 years of age or older; and (4) were available in full text in either English or French. Studies were not excluded based on year made available or publication status. Standardized criteria were developed to clarify the inclusion/exclusion criteria and to guide the selection of articles. Two independent reviewers applied the criteria to screen a random selection of 9% (*n* = 46) of the full text articles, which resulted in good inter-rater agreement (kappa = 0.784, SE = 0.119). Reviewer discrepancies were discussed until consensus was reached.

### Data extraction

Detailed data extraction instructions were developed to maintain consistency. The instructions clarified the data to be extracted, their possible locations in the articles, and the way to document them; illustrative examples were provided. Multiple reports of a single study were grouped together in the data extraction tables so that they would be counted once in the analysis, and so that all data about a single intervention was in one place.

Data extracted from all articles included name of first author, year published, and whether it was a peer-reviewed article, book chapter, or dissertation. For empirical quantitative or qualitative studies, the following additional information was documented: study design; contextual details (i.e. target population, country that the study was conducted in, cultural background of participants, and setting based on descriptions of the recruitment sites); and information about the sample (i.e. sample size, age, gender, level of cognitive impairment, marital status).

Data extracted from articles that described or evaluated an intervention also included the theoretical underpinnings of the intervention (i.e. any explicit guiding framework or theory, and the authors’ more implicit conceptualizations from the rationale provided for why the intervention may affect loneliness/social connectedness), and details of the intervention. Pertinent details of an intervention include its goal, components and activities, and mode of delivery and dose [28]. The goal referred to what the intervention was set to achieve. Active ingredients were the components and the activities required to implement each component, as operationalized in the study. Delivery mode was the format for giving the intervention, and dose was the amount of the intervention to which participants were exposed. Additional data extracted from qualitative studies that explored strategies to promote social connectedness among older adults were the results that identified and / or described strategies to affect loneliness/social connectedness, and supporting descriptions if needed to clarify the nature of the strategy.

### Analysis

Frequencies were calculated to describe the context (i.e. target populations and settings) in which the intervention was implemented. Strategies to affect loneliness/social connectedness extracted from qualitative studies were analyzed as follows: (i) an initial list of two indicators of social connectedness/loneliness (i.e., caring for and about others and feelings of belonging) and nine modifiable influencing factors (i.e., social network, social support, self-reported health, technology use, formal group memberships, mental and emotional well-being, community-based social participation, religious affiliation, and use of services such as home care) identified from a previous review [1] was used as the initial coding framework; (ii) the studies’ findings were coded and grouped together when similar (i.e. manifest content analysis), and new codes created as needed, thereby adding the factors of keeping busy/purposeful activity and personal development to the initial list; and (iii) the frequency of indicators and factors addressed across studies was assessed (i.e. metasummary [33]). The first author completed this analysis, and the last reviewed the findings.

To analyze the interventions, manifest content analysis was used to group similar types of interventions, and identify and describe their components. The first author completed this analysis and the last reviewed the findings. The first and second authors then independently assessed the extent to which the authors’ hypothesized mechanisms of effect (i.e., the theoretical underpinnings of the intervention) mapped to the indicators of and the list of factors that may influence social connectedness. Factors were not always explicitly stated in the articles, so this mapping involved making low-level inferences based on a careful review of the authors’ descriptions and definitions of hypothesized mechanisms of effect. Having two independent reviewers complete this analysis improved credibility of the findings. Discrepancies were resolved through discussion and review of coded data until consensus was reached on a final list of factors targeted by each intervention.

## Results

### Search results

**Fig. 1:**
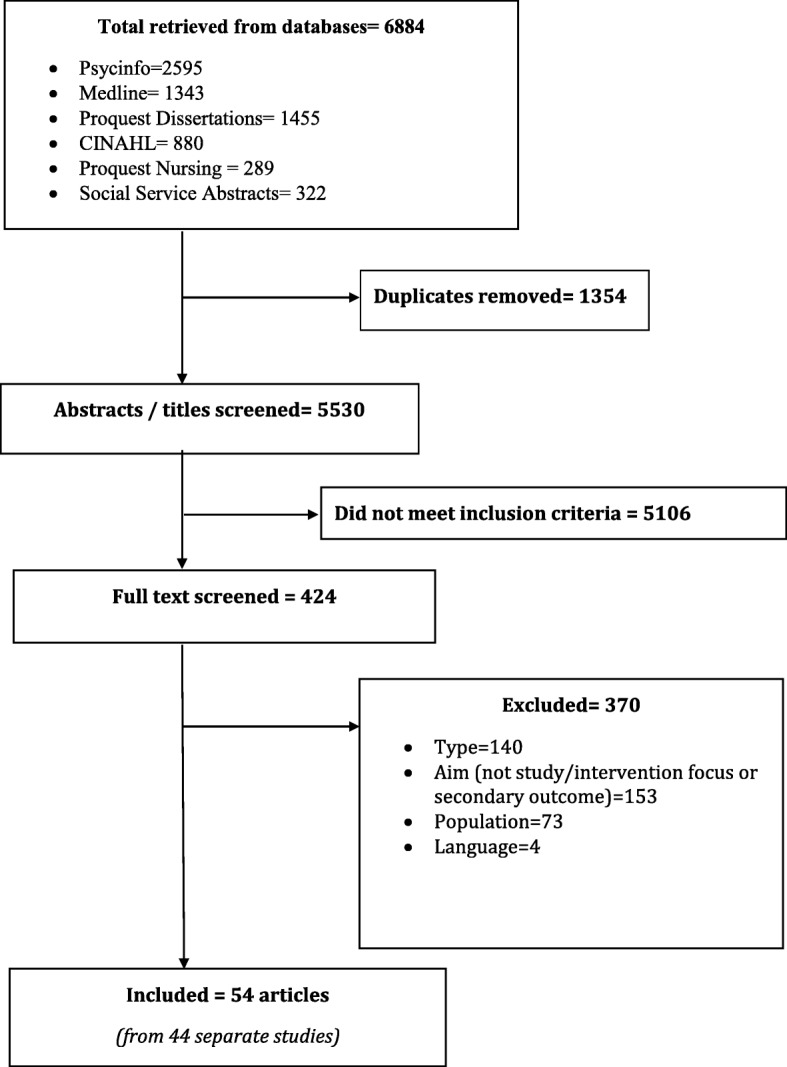
Search results. Results of the search and screening process

From 5530 titles/abstracts (the number after removal of duplicates), 424 full text articles were reviewed, and 44 separate studies (reported in 54 articles) met the inclusion criteria (Fig. 1). Studies were conducted primarily in the United States (38.6%) or in the Netherlands or other Nordic countries (22.7%). Most often, cultural background of the study sample or target population was not clearly reported (54.5%). Few studies included a majority of non-white participants (4.5%). Most of the studies focused on people who lived in the community, either independently, in assisted living, or ‘living alone’ (54.5%). The studies’ samples were often described as cognitively intact (31.8%) or level of cognitive function was not reported (31.8%), and usually included a majority (i.e. ≥ 50%) of women (86.4%) (Table 1). A few qualitative studies explored strategies to impact older adults’ loneliness/social connectedness from the perspective of older adults or their caregivers (11.4%); most of the studies described or evaluated an intervention (88.6%). Study details can be found in Additional file 2. Next, the synthesis of strategies from the qualitative studies is discussed, followed by the description of the core features of the interventions.

**Table 1:** Key characteristics of included studies (*N* = 44)

Characteristic	% (N)
Country	
United States	38.6% (17)
The Netherlands / Nordic countries	22.7% (10)
Australia/New Zealand	13.6% (6)
the United Kingdom	9.1% (4)
Asian countries	6.8% (3)
Canada	6.8% (3)
Eastern Europe	2.3% (1)
Culture	
Not reported	54.5% (24)
More than 60% white or Caucasian participants	40.9% (18)
More than 50% non-white participants	4.5% (2)
Setting	
Community *(*i.e. *Lived independently, in assisted living or ‘alone’)*	54.5% (24)
Residential care *(*i.e. *Nursing home or long term care facility)*	29.5% (13)
Mixed community/residential care	13.6% (6)
Not reported	2.3% (1)
Cognitive Impairment	
Cognitively intact	31.8% (14)
Not reported	31.8% (14)
Included some people with mild impairment	29.5% (13)
Majority cognitively impaired to a mild, moderate or severe degree	6.8% (3)
Gender	
50% or more of participants were women	86.4% (38)
Not reported	9.1% (4)
50% or more of participants were men	4.5% (2)

### Strategies from qualitative studies

The most common strategies described were engaging in purposeful activity or keeping busy (*n* = 5 studies) [6, 34–38] and having contact with a social network [6, 34–38] (n = 5). Purposeful activity/keeping busy was about engaging in pastimes or daily responsibilities, regardless of whether these involved contact with others and which were actually often solitary in nature, with the intent to pass the time or to accomplish something. Examples of activities included reading, listening to music, going for a walk, cleaning, cooking, and arts and crafts. All studies also described maintaining contact or interaction with a social network as an important strategy (*n* = 5) [6, 34–38]. Both in-person interactions and interactions occurring over the telephone or email were described. Of the five studies, three emphasized the importance of maintaining familiar relationships with family and friends, as opposed to making new contacts [6, 36, 37, 39].

Social participation, community-based activities that people choose and that have the potential to bring them into contact with others like volunteer work, joining clubs, or pursuing recreational activities [15], was discussed as a strategy in three studies [34, 35, 37–39]. One study described how people who had led active lives joined groups [34, 35], while another described how community-dwelling older adults watched television to feel connected to and engaged in the social world [37, 39]. Another study described how older adults who were part of a senior’s club joined groups, went on bus trips, went dancing, or attended a senior’s centre [38].

Just two studies explicitly described strategies that directly targeted the need to feel cared for, described as needing to have satisfying emotional relationships that result in feeling valued or affection from others [36] or seeking companionship from pets [37, 39]. All studies did emphasize having contact with *familiar* people (i.e. existing friends and family), which suggests that older adults may seek out the type of contact that results in feelings of being valued or cared for [6, 36, 37, 39].

Two studies described a strategy of personal development to change one’s outlook on life or social relationships. In one study, the people described how personal growth could result in reframing their outlook on solitude to prevent loneliness [38]. In the other, people would feel better about their own situation when comparing themselves to those perceived as being worse off or more lonely than they were [34, 35]. Social support, which refers to receipt of different types of assistance from others [9], was only explicitly described in one study [37, 39]. Interestingly, in this instance, it was not receiving social support, but providing social support that was described as a strategy to ameliorate loneliness, because helping others made people feel needed. Finally, having a religious affiliation and belief in a higher power, regardless of church attendance, was identified in one study to provide strength to combat loneliness [38].

### Interventions

Nine different intervention types, classified based on their components (i.e. active ingredients), were identified in 39 studies (Table 2). The mapping of theoretical underpinnings—the authors’ descriptions or rationale for why an intervention would work—to indicators and factors that influence loneliness/social connectedness, by intervention type across all studies, is shown in Table 3 and summarized in-text, following the descriptions of the interventions’ components. One-to-One Personal Contact (21%, *n* = 8) and Activity Group (18%, *n* = 7 studies) were the interventions that were most frequently explored or evaluated. In One-to-One Personal Contact, participants were paired with another individual, either a family member, friend or a volunteer for those with a more limited social network, and given the opportunity to have scheduled contact and interaction with that person. One-to-One Personal Contact was conceptualized as targeting five different indicators/factors, but most often social network (*n* = 8) and social support (*n* = 6), while caring, belonging, and technology use (e.g., phone, computer) were described in fewer than half of the studies.

**Table 2:** Intervention descriptions by type

Intervention Type	Components & Activities	Mode of Delivery	Doses Described
Personal Contact [17, 44–50] (8 studies/reports) Scheduled contact with another person.	1. Personal contact: Scheduled contact with someone from one’s social network (e.g. a family member or friend) or a volunteer who may be similar to the recipient on some characteristics (e.g. age, gender, interests, culture). Discussions are unscripted and informal in nature. Personal contacts may be specifically trained to offer emotional support, and may or may not be permitted to provide instrumental support (e.g., transportation, shopping, minor housekeeping and repairs, letter writing/correspondence, or meal preparation).	• One-to-one • Face-to-face, phone, email, or videoconference • Researcher supports training with videoconference equipment	• Usually weekly • 5 min to 1 h or more per interaction • 3 to 36 months
Activity & Discussion Groups [51–61] (7 studies, 11 reports) Engagement with a new group of people in an activity and/or facilitated discussion.	1. Group Activity Participation: joining a new group, and engaging in an activity of interest (e.g. song-writing, gardening projects, painting, pottery, dance, music, poetry, drama, jewelry making, storytelling, therapeutic writing, exercise, etc.) 2. Group Discussion: joining a new group, and engaging in facilitated discussion focused on a pre-determined topic of interest to the participants (e.g. movie discussion, bible discussion, role of the retiree) sharing of personal experiences. Group members may be taught how to offer emotional support to one another. All interventions of this type either addressed component 1 [57, 58], component 2 [54, 55, 57, 59–61], or both components 1&2 [51–53]	• Group of 5 to 9 people • Face-to-face sessions held in a neutral location (e.g. an activity room). • Facilitated (e.g. by a health care provider, researcher, university student, professional artist) who guides discussion and provides resources (e.g. songbooks, painting supplies).	• Sessions once or twice per week • 90 min to 6 h per session • 3 to 72 weeks
Animal Contact [62–68] (6 studies, 7 reports) Scheduled contact with an animal	1. Animal Contact: contact with an animal, usually a cat or dogs; animal simulations have also been used (e.g. a robotic seal). Activity with the animal is self-guided and may include talking to, holding, petting, playing with, grooming, or walking the animal on a leash. A person’s preference for animal type (e.g. cat or dog) as well as size (e.g. small or large) may be assessed in order to individualize the treatment. Sometimes, the person is assigned to one animal to have contact with for all sessions.	• Face-to-face • One-to-one in a person’s private room OR to a group (size has ranged from 2 to 13) in a common space of a long-term care facility (e.g. activity room, garden, etc.). • A pet attendant brings and supervises animals	• 1 to 3 times per week • 30 to 90 min per session • 3 to 24 weeks OR • Animal lives at the long term care or assisted living facility and residents can seek out contact
Skills Course [69–76] (6 studies, 8 reports) Courses delivered to develop participants’ personal skills.	1. Skills instruction: Instruction given to older adults to improve their ability to have contact with others and/or to enhance their existing friendships, or to make new friends. The activities differ depending on the type of skills that are taught. Skills taught have included: mindfulness-based stress reduction [76], use of a computer and internet [74, 75], those supporting friendship development (e.g. self-esteem, reflection on expectations of friendships, analysis of one’s network, goal and boundary-setting) [69–72], and self-management (to meet needs of comfort, stimulation, affection, behavioral confirmation and status [73]).	• Face-to-face • Usually group (size has ranged from 5 to 15) (one study was one-to-one [75]) • Lead by trained clinicians, researchers, experienced instructors	• Usually weekly • 2 to 4 h per session • 3 to 12 lessons • Some also include skills practice at home
Varied/Non-Specific [40, 41, 77–80] (5 studies, 6 reports) Broad, multifaceted programs not focused on a single type of intervention	1. Service delivery or social assistance: These are usually multi-component interventions, but the different components are not clearly conceptualized or described, and are described broadly as improving service delivery within a community. Types of services offered in a single program to a community may be related to any combination of: increased fitness or arts programs, or other social/leisure activities; improved transportation; access to information or resources; and/or consultation with medical, nursing, counseling, financial or housing experts.	Unclear as to who delivered different services and/or how these were delivered	• Programs lasted for 6 months to 2 years • Unclear what individuals within a community would have received
Model of Care [81, 82] (2 studies/reports) Implementation of a new philosophy for care provision (i.e. the Eden Philosophy)	1. Increase spontaneous, resident-driven and purposeful interaction with plants, animals and children: the Eden philosophy is about creating a ‘human habitat’ by providing opportunity for residents to interact as desired with plants, animals and children in their daily life in order to address issues related to loneliness, boredom and helplessness experienced by residents of long-term care facilities. Both interventions described staff training to understand the philosophy. One intervention also stated that work teams were formed to increase the number of plants, animals and involvement with children in the facility by opening a day-care program [81].	Unclear who was trained, and what implementation of this philosophy looked like in practice.	Unclear what facilities actually did to implement the philosophy
Reminiscence [83, 84] (2 studies/reports) Recalling and discussing past memories and experiences	1. Reminiscence: A topic or theme is provided for the session (e.g. school days, holiday traditions, first house and anniversaries, family histories and life stories, etc.) and participants share with the group their recollections.	• Face-to-face in a comfortable setting • Group (size not reported or ≤ 10) • Lead by facilitators trained in reminiscence therapy and who may have experience working with older adults	• 1 to 2 times per week • 1 to 1.5 h • 4 to 8 weeks
Support Group [85, 86] (2 studies/reports) Sharing personal challenges and receiving emotional or informational support.	1. Peer support group: Participants attend meetings where they select areas of discussion related to their needs and challenges that they face. Peer and professionals provide information resources and/or emotional support related to identified challenges.	• Face-to-face • Group (size range from 5 to 12) • Co-lead by peers who had strong interpersonal skills and professionals (e.g. nurse, minister, social worker, music therapist) experienced in working with older adults who face challenges (e.g. bereavement) or by staff who received training on the program components (e.g. activity aides)	• Weekly • 0.75 to 1.5 h • 12 to 20 weeks
Public Broadcast [87] (1 study/report) Listening to a generation-specific radio program.	1. Familiar music: Listening to music (primarily), serials and other segments of radio programs that were popular in the 1920–1950s. 2. Interactive style: programming delivered using a friendly and interactive style including birthday messages and ‘cheerio’ calls.	• to individuals • via a community radio station; free radio receiver provided, operated by a simple on/off switch	• Daily • 1 h • 3 months

**Table 3:** Theoretical underpinnings: proportion of studies targeting each modifiable^a^ factor and indicator, by type of intervention based on proposed mechanisms of action

	Intervention Type								
	Personal Contact [17, 44–50] (8 studies/reports)	Activity Group [51–61] (7 studies, 11 reports)	Animal Contact [62–68] (6 studies, 7 reports)	Skills Course [69–76] (6 studies, 8 reports)	Varied/Non-Specific [40, 41, 77–80] (5 studies, 6 reports)	Model of Care [81, 82] (2 studies/reports)	Reminiscence [83, 84] (2 studies/reports)	Support Group [85, 86] (2 studies/reports)	Public Broadcast [87] (1 study/report)
Proportion of studies targeting each factor/indicator									
Caring	3/8	3/7	4/6	3/6		2/2		1/2	1/1
Belonging	1/8	1/7							
Social Network	8/8	4/7	4/6	4/6	5/5	2/2	1/2		
Social Support	6/8	1/7	2/6	1/6	2/5			2/2	
Personal development		1/7		4/6	2/5		1/2	1/2	
Technology use	2/8			2/6					1/1
Busy/Purposeful			1/6	1/6					1/1
Mental/Emotional				1/6			1/2		
Social participation		6/7			1/5				

^a^There are additional influencing factors that are non-modifiable or difficult to modify. These include marital status, age, living arrangement, cognitive ability, sex or gender, level of formal education, income, religious affiliation, family composition, ethnicity, and death of a spouse. All modifiable factors were identified in a previous literature review [1], except for keeping busy/purposeful activity and personal development, which were identified in the current review from the synthesis of strategies identified in qualitative studies. Modifiable factors of service use, self-reported health and group memberships were not targeted by any of the studies

The components of Activity Group interventions included engaging in an activity of interest, and facilitated discussion aimed at promoting the development of relationships that may be characterized by support, friendship or companionship among group members. Many different activities were used, both within and across studies (e.g. arts and music, topical discussion, or exercise). Across the seven studies, Activity Groups were described as targeting six different indicators/factors including: caring, belonging, social support, and personal development (each described in less than half of the studies); and social participation (*n* = 6) and social network (*n* = 4) (each described in more than half of the studies).

Animal Contact (15%, n = 6), Skills Course (15%, n = 6), and Varied/Non-Specific (13%, *n* = 5) interventions were also explored in multiple studies. In Animal Contact interventions, most often referred to as ‘Animal Assisted Therapy’, participants were paired and allowed to interact with an animal, usually a dog or a cat, either at their own convenience or at a scheduled time. Animal Contact was described as targeting four indicators/factors, most often caring (*n* = 4) and social network (n = 4); social support and keeping busy/purposeful activity were also described, but in fewer than half of the studies. Skills Course interventions promoted the development of the person’s personal characteristics or skills in order to address loneliness/social connectedness. These involved training participants to obtain computer skills to increase access to resources or interpersonal contacts (*n* = 2), to develop psychological skills, like expectations of friends, self-esteem, or self-management, needed to develop and maintain strong interpersonal relationships (*n* = 3), or mindfulness skills to re-frame how one thinks about social relationships (*n* = 1). Skills Course interventions were described as targeting seven indicators/factors, but most often social network (*n* = 4), personal development (n = 4) and caring (n = 3); the others described in less than half of the studies included social support, technology use, keeping busy/purposeful activity, and mental and emotional well-being (e.g., avoiding stress).

Interventions that were classified as Varied/Non-Specific referred to the implementation, usually by a public health or community organization, of a large and multifaceted program that employed a combination of different types of interventions that were poorly specified. For example, the ‘Healthy Aging’ program [40, 41] was broadly conceptualized as addressing loneliness using any one of a variety of activities (e.g. reminiscence, skills course, or group activity). Each community involved in the program received a different type of intervention under the umbrella of ‘Healthy Aging’. Varied/Non-Specific interventions were conceptualized as addressing four factors, most often social network (*n* = 5), and social support, personal development and social participation in less than half of the studies.

Reminiscence (5%, *n* = 2), Support Group (5%, n = 2), Model of Care (5%, n = 2), and Public Broadcast (3%, *n* = 1) were each explored or evaluated in just one or two studies. Reminiscence referred to an intervention where the primary component was to prompt participants to reflect on past memories or experiences. One of these interventions also had participants consider the future in light of insights gained, while the other described communication facilitation techniques aimed at promoting positive interactions between participants in a group setting. Reminiscence was conceptualized as targeting three factors: social network (*n* = 1), personal development (n = 1), and mental and emotional well-being (n = 1).

Support Group interventions involved bringing together a group of people who shared some common characteristics or life experiences, and facilitating group members to provide emotional, informational, or appraisal support through exploration of issues that members experienced. Support Groups differed from Activity Groups in that the focus was on the promotion of social support, not on engagement in an activity or building other types of relationships with others. Support Groups were described as targeting the indicator of caring (*n* = 1) and the factors of social support (*n* = 2) and personal development (n = 1).

Model of Care interventions implemented a philosophy of care within a long-term care facility that was clearly described as targeting loneliness/social connectedness as a primary outcome. Both studies identified in this review described implementation of the Eden Alternative Model philosophy, which espoused development of close relationships between staff and long-term care residents, the integration of plants and animals into the everyday lives of residents, and promoting residents’ active involvement in the day-to-day life at the long-term care facility. Model of Care interventions targeted the indicator of caring (*n* = 2) and the factor of social network (n = 2). The final intervention type was a Public Broadcast. In this intervention, participants were asked to listen to a radio program that played music that may be familiar to participants based on their age, was presented by a friendly radio announcer, and included some programming that used an interactive style. The Public Broadcast intervention targeted the indicator of caring (*n* = 1), technology use (n = 1), and keeping busy/purposeful activity (n = 1).

## Discussion

This review identified nine distinct intervention types, classified based on their active ingredients, developed to address loneliness/social connectedness among older adults. Within each of the nine types of interventions, the studies’ authors had different theories about what factors were targeted, signaling inconsistency in the literature regarding the mechanisms by which the interventions have been conceptualized to affect loneliness/social connectedness. In addition, authors frequently conceptualized a single component of an intervention (e.g. contact with a relative or visitor) as targeting multiple factors (e.g. caring, belonging, social network, social support), but did not test the assumption that a single component affected multiple outcomes. Taken together, these two features of the literature make it unclear whether an intervention component really accomplishes all that it is theorized to. Research that would strengthen the evidence base includes theory-informed intervention evaluation that delineates the series of factors that are targeted by the intervention as immediate outcomes and examines effects of the intervention on each factor [28].

Findings from this review supported that the types of interventions that have been developed to date address, in theory, many of the key strategies identified by key stakeholders including contact, social participation, feeling cared for, personal development and social support [6, 34–38]. Contact, or development of one’s structural social network, was described as a target in all but two intervention types (the exceptions were ‘Social Support’ and ‘Public Broadcast’). Social participation was described as a target in 86% of ‘Activity Group’ interventions. Feeling cared for was described as a target in 67% of ‘Animal Contact’ and 100% of ‘Model of Care’ interventions. Personal development was described as a target in 67% of ‘Skills Course’ interventions, and social support was described as a target in 75% of Personal Contact and 100% of Social Support interventions. Of note, none of the interventions directly targeted the strategy of purposeful activity/keeping busy, the importance of which was emphasized by key stakeholders in all five qualitative studies [6, 34–38]. Development of interventions to address loneliness/social connectedness which target purposeful activity/keeping busy would address stakeholder priorities and is consistent with calls to develop a range of interventions that are responsive to characteristics of participants and to their needs and preferences [42].

Other reviews have found highly inconsistent results of intervention impact within each of the different categories of interventions, concluding that there was very limited evidence supporting effectiveness of the interventions [21–24]. However, there are two key conceptual issues that may have affected these reviews’ findings [43]: (1) mixing the findings of effectiveness of heterogeneous interventions, which will lead to inconsistent findings across studies; and (2) analyzing studies of an intervention delivered using a single mode of delivery, which will lead to an incomplete synthesis. The categories of interventions identified in the present review address the above issues in two ways and may be useful to guide future systematic reviews to establish efficacy. First, interventions were only analyzed if they targeted loneliness/social connectedness (not other social concepts like isolation/contact). Second, interventions were grouped according to their active ingredients, not the mode of delivery. While two of the intervention types referred to in the present review do specify whether the activity occurred in a group (i.e. Group Activity) or one-to-one (i.e. One-to-One Personal Contact), in these instances, group and one-to-one delivery represent an active ingredient (i.e. an element of the intervention hypothesized to affect the outcome) rather than simply a mode of delivery (i.e. how participants are given the intervention). Other intervention types described in the present review (e.g. Animal Contact) could be delivered in a group or to an individual. Mode of delivery should be considered in reviews of intervention effectiveness, but may be more appropriately examined as a secondary aim (e.g. is Animal Contact as effective when delivered in a group or one-to-one?), rather than as a primary aim (e.g. are interventions delivered to groups effective?).

Limitations of this study included that only studies in English and French were reviewed; other interventions and strategies have likely been described but the reports made available in other languages. Strengths of the review included a comprehensive search, transparent and standardized data extraction and screening procedures, in-depth description and synthesis of data, and independent reviewers for coding the interventions’ theoretical underpinnings, which was the most subjective part of the analysis.

## Conclusion

The findings from this review identified the interventions that have been designed to address loneliness/social connectedness for older adults, and described their key features as well as the influencing factors that they have been conceptualized to target. As such, the findings provide a strong foundation for future research to advance theory-informed design and evaluation of interventions to affect loneliness/social connectedness. The interventions identified also aligned with many of the strategies that have been used by older adults or caregivers to address loneliness/social connectedness among older adults, highlighting their relevance to key stakeholders. Future research should include systematic reviews to examine effectiveness of the distinct types of interventions that target loneliness/social connectedness, studies to test the interventions’ theorized mechanisms of action, and development of interventions that target purposeful activity as a way to ameliorate loneliness among older adults.

## Additional files

Additional file 1: Data-base specific search strategies. Provides the full search strategy for each database. (DOCX 18 kb)

Additional file 2: Study details. Provides detailed information of each study including: design, context (i.e., target population, country, culture, setting), and sample characteristics (i.e., sample size, age, gender, cognitive impairment level, and marital status). (DOCX 46 kb)
