# Supplementary material for: Interventions to address social connectedness and loneliness for older adults: a scoping review
Source: BMC Geriatr. 2018 Sep 15;18:214. doi: 10.1186/s12877-018-0897-x (PMC6139173; doi:10.1186/s12877-018-0897-x)
Supplement: Supplementary file 1 — Data-base specific search strategies. Provides the full search strategy for each database. (DOCX 18 kb) [file 12877_2018_897_MOESM1_ESM.docx]

Additional file 1. Database-specific search strategies

**Psycinfo**

((“social* connected*” or connectedness or lonely or loneliness or “feel* alone*”) OR su.exact("loneliness") OR su.exact("Social Isolation ")) AND (trial* OR intervention* OR therapy OR evaluat* OR implement* OR (qualitative AND strateg*) or su.exact(“Intervention”))

Limits:

Age group: Adulthood (18 Yrs & Older)

**Medline***

1. Intervention Studies/

2. (trial* or intervention* or therapy or evaluat* or implement* or (qualitative and strateg*)).mp.

3. Loneliness/

4. Social Isolation/

5. limit 4 to yr="1861 - 1976"

6. ('social* connected*' or connectedness or lonely or loneliness or 'feel* alone*').mp.

7. 1 or 2

8. 3 or 5 or 6

9. 7 and 8

10. limit 9 to "all adult (19 plus years)"

**CINAHL***

1. (MH "Experimental Studies+") OR (MH "Intervention Trials")
2. (trial* or intervention* or therapy or evaluat* or implement* or (qualitative and strateg*))
3. 1 OR 2
4. (MH "Loneliness")
5. (MH "Social Alienation")
6. ("social* connected*" or connectedness or lonely or loneliness or "feel* alone*")
7. 4 OR 5 OR 6
8. Limiters - Age Groups: All Adult

**Proquest Dissertations and Thesis**

all(trial* OR intervention* OR therapy OR evaluat* OR implement* OR (qualitative AND strateg*)) AND all("social* connected*" OR connectedness OR lonely OR loneliness OR "feel* alone*")

**Proquest Nursing**

all(trial* OR intervention* OR therapy OR evaluat* OR implement* OR (qualitative AND strateg*)) AND all("social* connected*" OR connectedness OR lonely OR loneliness OR "feel* alone*")

Age group limit: Adult (19-44 years) or Aged (65+ years) or Aged (80+ years) or Middle aged (45-64 years)

**Proquest Social Service Abstracts**

(trial* OR intervention* OR therapy OR evaluat* OR implement* OR (qualitative AND strateg*)) AND ("social* connected*" OR connectedness OR lonely OR loneliness OR "feel* alone*")

*****The scope notes in these databases clearly differentiate between Loneliness (an unpleasant feeling or experience) and Social Isolation (absence of contact with others) in article indexing (i.e., use of subject or MESH heading tags). In these situations, the Social Isolation SH or MESH was not included in the search strategy, as it would result in a large number of records of studies that would not meet the inclusion/exclusion criteria (i.e. about contact, not connectedness-loneliness). Other databases did not support use of SH or MESH (keyword searching only) at the time when the search was conducted.
