# Supplementary material for: Interventions to address social connectedness and loneliness for older adults: a scoping review
Source: BMC Geriatr. 2018 Sep 15;18:214. doi: 10.1186/s12877-018-0897-x (PMC6139173; doi:10.1186/s12877-018-0897-x)
Supplement: Supplementary file 2 — Study details. Provides detailed information of each study including: design, context (i.e., target population, country, culture, setting), and sample characteristics (i.e., sample size, age, gender, cognitive impairment level, and marital status). (DOCX 46 kb) [file 12877_2018_897_MOESM2_ESM.docx]

Additional file 2. Study details

| **First Author (year)** | **Design** | **Context** | **Sample Characteristics** |
| --- | --- | --- | --- |
| Andersson (1984, 1985) | Quasi-experimental, non-randomized, controlled | Target population: older women at risk for loneliness—living alone, on a wait list for institutional care, and who stated were lonely  Country: Sweden  Culture: NR  Setting: an urban setting | Sample Size: 108 [68 intervention, 40 control]  Age: M= 77; Range=60-80  Gender: 100% women  Cognitive Impairment: NR  Marital Status: Single=24%, Divorced=14%, Widowed=63% |
| Andrews (2003) | Qualitative exploratory descriptive | Target population: frail and isolated older people  Country: United Kingdom  Culture: Most of white background (% NR)  Setting: users of a befriending program who lived alone in ‘their own homes’ | Sample Size: 13  Age: M= 86.5; Range NR  Gender: 77% women  Cognitive Impairment: NR  Marital Status: NR |
| Baker (2013) | Pilot study, uncontrolled, qualitative exploratory | Target population: retirees without cognitive impairment and varied musical backgrounds  Country: Australia  Culture: 100% of Anglo-Celtic origin  Setting: independent living units in a retirement village | Sample Size: 8  Age: M= 80.5 (SD=12.3); Range NR  Gender: 75% women  Cognitive Impairment: no diagnosis of dementia or generalized cognitive deterioration  Marital Status: NR |
| Banks (1998, 2002) | Randomized controlled trial | Target population: long term care residents  Country: United States of America  Culture: 91% white; 9% African American  Setting: three long term care facilities in a Southern city | Sample Size: 45 [15 control; 15 low exposure; 15 high exposure]  Age: M= NR; Range=55-95 (older than 75=71%)  Gender: 80% women  Cognitive Impairment: free from any cognitive impairment as stated by the physician in the history and physical examination  Marital Status: Married=9%; Single=4%; Divorced/Separated=9%; Widowed=78% |
| Banks (2005) | Randomized controlled trial | Target population: lonely long term care residents  Country: United States  Culture: 84% white; 16% black  Setting: three long term care facilities in St. Louis, Missouri; some lived in assisted living portion of the facility | Sample Size: 37 **[**17 individual therapy, 20 group therapy]  Age:  Group 1 (n=17): mean= 83.2 ± 5.4; Range= 69–90  Group 2 (n=20): mean= 81.1 ± 4.5; Range= 69–87  Gender: 59% women  Cognitive Impairment: all cognitively intact (> 24 on the modified MMSE); no diagnosis of Alzheimer’s disease  Marital Status: NR |
| Bartlett (2013) | Two pilot projects, single group pre/ post evaluation | Target population: older people at risk of social isolation and loneliness— culturally and linguistically diverse communities in rural or remote locations  Country: Australia  Culture: NR  Setting: recruited through community organizations living alone, with partner/family, with carer or in some other arrangement | Sample Size: 58  Age:  Group 1 (n=16): Mean=79 (SE1.1); Range=63-100  Group 2 (n-42): Mean=66 (SE 1.2); Range 54-93  Gender: 59% women  Cognitive Impairment: NR  Marital Status: NR |
| Bergman-Evans (2004) | Quasi-experimental, controlled pre/post test | Target population: long term care facility residents  Country: United States  Culture: 92% white, 14% non-white  Setting: veterans’ home in a Midwestern city | Sample Size: 64 [35 experimental, 29 control]  Age:  Group 1 (n=35): mean=76.1 (SD=13); Range=51-105  Group 2 (n=29): mean=83.1 (SD=5.8); Range=75-94  Gender: 59% women  Cognitive Impairment: cognitively intact  Marital Status: NR |
| Blazun (2012) | Quasi-experimental, pre/ post test | Target population: older people  Country: Finland & Slovenia  Culture: NR  Setting: recruited from the community at large and two elder care homes [98% urban, 2% rural], | Sample Size: 45  Age:  Finland (n=17): M= 66.34; SD = 6.0; Range: 57+  Slovenia (n=28): M=77.36; SD=8.24; Range: NR  Gender: 62% Women  Cognitive Impairment: NR  Marital Status: NR |
| Brown (2004) | Experimental, wait-list control group pre/post test | Target population: elderly nursing home residents  Country: United States  Culture: 82% white**,** 18% black  Setting: rural nursing homes in the southern states | Sample Size: 66  Age: M= 81, Range=60-96  Gender: Women=82%  Cognitive Impairment: could understand all questions and respond verbally or in writing  Marital Status: Married=14%, Single=6%, Divorced=9%, Widowed=71% |
| Cattan (2003a, 2003b) | Case study | Target population: lonely or socially isolated older people  Country: United Kingdom  Culture: from a range of social and geographical backgrounds  Setting: the majority lived alone | Sample Size: 193 [23 staff, 25 older people (individual interviews) and 145 older adults (focus groups)]  Age: M= NR; older people were between 55-94  Gender: NR  Cognitive Impairment: NR  Marital Status: majority widowed |
| Cattan (2011) | Exploratory qualitative | Target population: vulnerable isolated and/or lonely older people  Country: United Kingdom (England and Scotland)  Culture: NR  Setting: most lived alone | Sample Size: 40 [27 service recipients, 6 volunteers and 7 recipients/volunteers]  Age: mean=NR; Range mid-50s to early 90s  Gender: NR  Cognitive Impairment: NR  Marital Status: NR |
| Chiang (2010) | Randomized controlled trial | Target population: institutionalized elderly people  Country: Taiwan  Culture: All spoke Mandarin or Taiwanese  Setting: nursing home institution in the Taipei area | Sample Size: 92 [45 experimental**,** 47 control]  Age: M= 77.24 (SD 3.97)  Gender: 100% men  Cognitive Impairment: Mini-Mental State Exam M= 23.10 + 1.98; all > 20 (intact cognitive function to mild impairment)  Marital Status: Married=15%, Single=58%, Divorced/separated=12%, Widowed=15% |
| Clarke 1992 | Pragmatic randomized controlled trial | Target population: elderly people living alone  Country: England  Culture: NR  Setting: a town in central England | Sample Size: 523 [261 experimental, 262 control]  Age: M= NR; all over 75 years  Gender: NR  Cognitive Impairment:  Score of 12/12 on the Clifton Assessment Procedures for the Elderly= 73%  Marital Status: NR |
| Cohen (2006a, 2006b) | Quasi-experimental, controlled pre-test double post-test | Target population: English-speaking community-based older adults  Country: United States    Culture: 30% racial and ethnic minorities  Setting: all living independently | Sample Size: 300 [150 intervention, 150 control]  Age: M~ 80, Range 65-100  Gender: ~78% women  Cognitive Impairment: NR  Marital Status: NR |
| Cohen-Mansfield (2007) | Qualitative exploratory | Target population: older individuals  Country: United States  Culture: NR  Setting: three low-income senior independent living housing buildings (2 government owned and one church owned) in suburban Maryland | Sample Size: 276  Age: NR  Gender: NR  Cognitive Impairment: NR  Marital Status: NR |
| Creswell (2012) | Randomized controlled trial | Target population: healthy older adults not currently practicing any mind–body therapies more than once per week  Country: United States  Culture: All English-speaking,  64% Caucasian, 12% African American, 10% Latino, 7% Asian American, and 5% Other  Setting: NR but likely community-based sample (recruited via newspaper ads) | Sample Size: 40 [20 treatment, 20 control]  Age: M= 65 (SD 7); Range 55–85  Gender: 83% women  Cognitive Impairment: > 23 on the Mini-Mental State Exam (intact cognitive function to mild impairment)  Marital Status: NR |
| Dammemeyer (2004) | Matched groups quasi-experimental, wait-list control | Target population: lonely elderly residents of assisted living facilities  Country: United States  Culture: NR  Setting: assisted living facilities | Sample Size: 34 [17 treatment, 17 control]  Age: M= 83.7 (SD 5.23)  Gender: 91% women  Cognitive Impairment: Temporal Orientation Test scores indicate ‘some cognitive impairment’**;** all were cognitively and behavioral appropriate and could attend to a conversation  Marital Status: NR |
| De Vlaming (2010)  Honigh de Vlaming (2013) | Quasi-experimental controlled pre-test post-test with qualitative process evaluation | Target population: non-institutionalized elderly people  Country: the Netherlands  Culture: Dutch people; 97% born in the Netherlands  Setting: a rural village in the eastern part of the Netherlands.  30% lived alone | Sample Size: 1802 **(**905 intervention**,** 897 control); 17 for the process evaluation (14 respondents + 3 of their spouses)  Age: 65-75=51%; >75=45%  Gender: 56.5% women  Cognitive Impairment: NR  Marital Status: Married=66.0%, Unmarried=3.5%, Divorced=3.5%, Widowed=27.5% |
| Fokkema (2007) | Quasi-experimental controlled prestest posttest | Target population: seniors at risk for loneliness, who not yet a computer or Internet user, but with no negative attitudes towards and the ability to work with a computer  Country: the Netherlands  Culture: Dutch  Setting: 73.1% lived alone | Sample Size: 26 [12 intervention, 14 control]  Age: Intervention M=66; 7 participants over 65  Control M= 68; 9 over 65 years  Gender: 69.2% women  Cognitive Impairment: NR  Marital Status: 1 person married in the intervention group; NR in the control group or overall sample |
| Hooper (2003) | Descriptive, exploratory qualitative | Target population: older adults with low levels of loneliness who are part of a senior’s club  Country: Canada  Culture: English speaking,  Catholic (53%), Protestant (20%), other faith (27%)  Setting: community dwelling who attend a community senior’s centre; living within 40 Km of Halifax; 93% lived alone in apartments | Sample Size: 15  Age: M= 77.2 (SD 7.7); Range 65-88  Gender: 80% women  Cognitive Impairment: not cognitively impaired  Marital Status: Married=0%, Single=0%, Divorced=3%, Separated=1%, Widowed=11% |
| Kremers (2006) | Randomized controlled trial | Target population: single community- dwelling women who missed having people around them, wished to have more friends, participated in very few leisure activities, or had trouble in initiating activities.  Country: the Netherlands  Culture: NR, but assume Dutch  Setting: community-dwelling women | Sample Size: 142 [63 intervention, 79 control]  119 completed [46 intervention, 73 control]  Age: NR at baseline; for those that completed intervention M=62.8 (SD 6.4), control M= 65.2 (SD 7.6)  Gender: 100% women  Cognitive Impairment: NR  Marital Status: NR at baseline; for those that completed intervention  Never married=17.6%, Divorced=46.2%, Widowed=37.0% |
| Martin (2006) | Pre-test/post-test follow-up control group design | Target population: older women  Country: the Netherlands  Culture: NR, but assume Dutch  Setting: community-dwelling, 67% single household, 28% couple, 4% partner& child(ren), 2% other | Sample Size: 115 [60 intervention, 55 control]  Age: M= 63  Gender: 100% women  Cognitive Impairment: NR  Marital Status: Married=31%, Never Married=7%, Divorced=32%, Widowed=30% |
| Moses (2003) | Case study methodology; un-controlled before after test | Target population: elderly citizens who had minimal impact with friends or relatives and were identified as suffering from depression or loneliness  Country: United States  Culture: 91% African Americans, 9% European American  Setting: lived in a nursing home or independent living facility in a low-income community in Detroit; 44% lived with spouse, 33% lived alone, and 22% with spouse + other family | Sample Size: 9  Age: Range 60-74  Gender: 67% women  Cognitive Impairment: did not have dementia  Marital Status: NR |
| Moyle (2011) | Descriptive exploratory qualitative | Target population: people with early-stage dementia, living in community and long term care  Country: Australia  Culture: NR  Setting: ten people with dementia recruited from community and 60 from long term care | Sample Size: 143 [70 people with dementia, 73 family carers]  Age: Range 66-97 people with dementia, 37-89 family carers  Gender: 70% women (people with dementia), 74% women (family carers)  Cognitive Impairment: early stage dementia, caregivers NR  Marital Status: NR |
| Ollonqvist (2008) | Randomized controlled trial | Target population: frail older people  Country: Finland  Culture: NR  Setting: community- dwelling; trial implemented in seven independent rehabilitation centres and 41 municipalities. 72% lived alone | Sample Size: 708 [343 intervention, 365 control=365]  Age: M= 78; Range 65-96  Gender: 86% women   - Cognitive Impairment: - People with Mini-Mental State Exam score < 18 points (moderate to severe impairment) were excluded   Marital Status: Widowed=63% |
| Parsons (2004) | Controlled before after study | Target population: nursing home residents  Country: United States  Culture:all English-speaking,  White=53%, Black=57%, Hispanic=10%, Other=10%  Setting: nursing home | Sample Size: 60 [27 intervention, 33 control]  Age: Intervention M= 76 (SD 10.8); Control M= 79 (SD 11.9)  Gender: 73% women  Cognitive Impairment: all scored less than 2 on the Minimum Data Set-Cognitive Performance Scale at baseline (intact or mildly impaired)  Marital Status: Married=7%, Never Married=17%, Divorced=10%, Widowed=67% |
| Passmore (2007) | One-group pretest double post test | Target population: individuals with early Alzheimer’s disease, behavioral disturbances who reported experiencing loneliness and decreased socialization or feelings of usefulness  Country: United States  Culture: NR  Setting: 53% Living with spouse, 27% other family, 17% in an institution, or 3% alone | Sample Size: 30  Age: Range 63-76  Gender: 57% Women  Cognitive Impairment: diagnosed with Stage 3 mild cognitive decline (early-stage Alzheimer’s Disease)  Marital Status: NR |
| Pettigrew (2008) | Qualitative exploratory | Target population: older individuals  Country: Australia  Culture: NR  Setting: independent living in either an independent dwelling (84%) or retirement village (16%) | Sample Size: 19  Age: M= 79; Range 65-95  Gender: 68% women  Cognitive Impairment: people with dementia excluded  Marital Status: Married=16%, Widowed/single=84% |
| Reed (1986) | Single-group equivalent time samples [0 X 0 X] | Target population: elderly nursing home residents  Country: United States  Culture: Roman Catholic nursing home, understood or spoke English  Setting: one nursing home | Sample Size: 48  Age: M= 82.9; Range 65-95  Gender: 92% women  Cognitive Impairment: two subjects (4%) had dementia and two others had organic brain syndrome  Marital Status: Married=2%, Never married=27%, Widowed=71% |
| Robinson (2013) | Randomized Controlled Trial | Target population: nursing home residents  Country: New Zealand  Culture: NR  Setting: retirement home, in the hospital and rest home areas, which provide 24-7 support | Sample Size: 40 [20 intervention, 20 control]  Age: Range 55-100  Gender: 68% women  Cognitive Impairment: Nineteen participants (48%) scored 6 or lower on the Abbreviated Mental Test, which is suggestive of cognitive impairment.  Marital Status: NR |
| Routsalo (2009)  Savikko (2009)  Pitkala (2009) | Randomized Controlled Trial | Target population: community dwelling older people suffering from loneliness at least sometimes  Country: Finland    Culture: NR  Setting: seven day care centers, excluded if living permanently in institutional care; 80% living alone | Sample Size: 235 [117 intervention, 118 control]  Age: M=80 years; Range=75-92  Gender: 74% women  Cognitive Impairment: 11% with Mini-Mental State Exam score <24 (impaired cognition); Range 19-30 (mild impairment to intact cognitive function)  Marital Status: Married=18%, Unmarried/divorced=14%, Widowed=69% |
| Schwindenhammer (2014) | Quasi-experimental within-between repeated measures design | Target population: nursing home elders  Country: United States  Culture: could all read and speak English  Setting: five Medicare/Medicaid nursing homes in central Illinois | Sample Size: 80 [40 intervention, 40 control]  Age: M= 86.12 (SD 5.28), Range 71-97  Gender: 88% women  Cognitive Impairment: Mini-Mental State Exam M= 23.9 (SD=1.83); Range 22-29 (mild impairment to cognitively intact)  Marital Status: NR |
| Smith (2011)  Smith (2012) | Interpretive phenomenology | Target population: community-dwelling older adults who had experienced loneliness in the last 6 months  Country: United States  Culture: 100% Caucasian  Setting: community-dwelling | Sample Size: 12  Age: Range 74-98  Gender: 67% women  Cognitive Impairment: Scored < 12 on the Short Blessed Test of Memory Orientation and Concentration (cognitively intact)  Marital Status: Married=25%, Single=25%, Widowed=50% |
| Stevens (2000)  Stevens (2001) | Matched case control | Target population: very lonely older women  Country: the Netherlands  Culture: NR  Setting: 75% living alone | Sample Size: 64 [32 program participants; 32 matched control]  Age: M= 63.2; Range 54-80  Gender: 100% women  Cognitive Impairment: NR  Marital Status: 25% Married, 9% Never Married, 28% Divorced, 38% Widowed |
| Stevens (2006) | Comparative observational correlational | Target population: very lonely older women  Country: the Netherlands  Culture: NR  Setting: 66% living situation alone, 29% with partner | Sample Size: combined intervention=112 (52 from study 1, 50 from study 2)  Control group (from study 2)=55  Dutch Aging Survey Comparison Group=226  Age: M=63.2; Range 53-78  Gender: 100% women  Cognitive Impairment: NR  Marital Status: Married=30%, Never married=7%, Divorced=30%, Widowed=33% |
| Stewart (2001) | Pretest, posttest, and delayed posttest within subjects design | Target population: widowed seniors  Country: Canada  Culture: all able to speak and write English  Setting: 86% lived alone | Sample Size: 23  Age: Range 54-77  Gender: 100% women  Cognitive Impairment: no neurological deficits  Marital Status: 100% widowed; number of years married varied from 6 to 53 years (M= 40.2 years) |
| Theurer (2014) | Mixed-methods qualitative process evaluation design | Target population: residents of long term care homes able to communicate verbally in a group discussion  Country: Canada  Culture: NR  Setting: three long term care homes | Sample Size: 72 [65 residents, 7 staff]  Age: 55% >85 ; 6% < 64  Gender: 83% women  Cognitive Impairment: 65% had dementia based on the Mini-Mental State Exam: 9% < 10, 46% 11-20, 34% 21-26, 11% 27-30  Marital Status: NR |
| Traves (2011) | Single group pre-test post-test | Target population: community-dwelling persons and residents of residential care facilities, aged 60 years and older  Country: Australia  Culture: could speak or understand English  Setting: 59% lived in the community with 61% living alone; 40% lived in a residential care facility | Sample Size: 113  Age: M= 79.9 years (SD 8.9); all older than 60  Gender: 70.8% women  Cognitive Impairment: Mini-Mental State Exam M= 28.2 (SD 2.3); Range 19–30  5 people with mild or moderate dementia  Marital Status: NR |
| Tsai 2010 | Quasi-experimental controlled double post-test | Target population: nursing home residents  Country: Taiwan  Culture: NR  Setting: 14 medium to large nursing homes | Sample Size: 57 [24 intervention, 33 control]  Age: all older than 60, Intervention M= 74.4 (SD 10.2); Control M= 78.5 (SD 6.8]  Gender: 57.9% women  Cognitive Impairment: “Good” cognitive status with Mini-Mental State Exam Intervention M= 22.9 (4.0) and Control M= 22.2 (3.9) (means align with mild cognitive impairment)  Marital Status: Married= 35.1%, Single= 3.5%, Divorced= 1.8%, Widowed= 59.6% |
| Tsai 2011 | Longitudinal cluster randomized control trial | Target population: older nursing home residents  Country: Taiwan  Culture: NR  Setting: 16 nursing homes | Sample Size: 90 [40 intervention, 50 control]  Age: all older than 60, Intervention M= (SD ); Control M= (SD ]  Gender: 57.8% women  Cognitive Impairment: “Good” cognitive status with Mini-Mental State Exam Intervention M= 23.5 (4.0) and Control M= 22.2 (4.0) (means align with mild cognitive impairment)  Marital Status: Married=31.1%, Single= 3.3%, Divorced= 4.4%, Widowed= 61.1% |
| van der Heide (2012) | Single group pre-post | Target population: elderly home care recipients  Country: the Netherlands  Culture: Dutch  Setting: living independently and receiving home care; 83.2% lived alone, 16.1% lived with someone else | Sample Size: 130  Age: M=73.2 (11.8); Range 32-90  Gender: 69.8% women  Cognitive Impairment: people with severe cognitive deficits were excluded  Marital Status: NR |
| Van Orden (2013) | Randomized controlled trial (protocol paper) | Target population: older adults not residing in assisted living or skilled nursing facilities  Country: United States  Culture: English-speaking, 75% white  Setting: community-dwelling people recruited from primary care practices and community-based agencies | Sample Size: planned 400 [200 intervention, 200 control]  Age: over 60  Gender: 50% women (will be stratified by gender)  Cognitive Impairment: Low likelihood of dementia based on Mini-Cog score > 3  Marital Status: to be assessed in full study |
| Vrbanac (2013) | Single group before/after | Target population: geriatric nursing home residents  Country: Croatia  Culture: NR  Setting: a nursing home | Sample Size: 21  Age: M= 80.5 (6.6);  Gender: 80% women  Cognitive Impairment: NR  Marital Status: NR |
| Winstead (2014) | Case controlled before/after | Target population: older adults in assisted and independent living communities  Country: United States  Culture: 96% white  Setting: 15 assisted and independent living communities in a medium-sized metropolitan city | Sample Size: 141 [104 cases, 37 controls]  Age: M= 83.2 (SD 7.4)  Gender: 80.9% women  Cognitive Impairment: Not cognitively impaired  Marital Status: Married=12.8%, Divorced/Separated/Never married=17.7%, Widowed=69.5% |
| Wright (1995) | One group, pretest-postest | Target population: people 65 or older who were long term care facility residents for < 5 years  Country: United States  Culture: NR  Setting: an 83-bed long term care facility in East-Central Mississippi | Sample Size: 13  Age: M= 83.9; Range 71-91  Gender: 85% women  Cognitive Impairment: alert and oriented  Marital Status: Married=8%, Single=15%, Divorced=23%, Widowed=54% |
